# Connections Between Amino Acid Metabolisms in Plants: Lysine as an Example

**DOI:** 10.3389/fpls.2020.00928

**Published:** 2020-06-19

**Authors:** Qingqing Yang, Dongsheng Zhao, Qiaoquan Liu

**Affiliations:** ^1^Jiangsu Key Laboratory of Crop Genomics and Molecular Breeding, Key Laboratory of Plant Functional Genomics of the Ministry of Education, College of Agriculture, Yangzhou University, Yangzhou, China; ^2^Key Laboratory of Crop Genetics and Physiology of Jiangsu Province, Jiangsu Co-Innovation Center for Modern Production Technology of Grain Crops, Yangzhou University, Yangzhou, China

**Keywords:** amino acids, metabolic connection, lysine metabolism, stress responses, tricarboxylic acid cycle, tryptophan metabolism

## Abstract

Extensive efforts have been made to fortify essential amino acids and boost nutrition in plants, but unintended effects on growth and physiology are also observed. Understanding how different amino acid metabolisms are connected with other biological pathways is therefore important. In addition to protein synthesis, amino acid metabolism is also tightly linked to energy and carbohydrate metabolism, the carbon-nitrogen budget, hormone and secondary metabolism, stress responses, and so on. Here, we update the currently available information on the connections between amino acid metabolisms, which tend to be overlooked in higher plants. Particular emphasis was placed on the connections between lysine metabolism and other pathways, such as tryptophan metabolism, the tricarboxylic acid cycle, abiotic and biotic stress responses, starch metabolism, and the unfolded protein response. Interestingly, regulation of lysine metabolism was found to differ between plant species, as is the case between dicots and monocots. Determining the metabolic connection between amino acid metabolisms will help improve our understanding of the metabolic flux, supporting studies on crop nutrition.

## Introduction

Amino acids play a number of vital roles in the central metabolism of plants. Essential amino acids (EAAs), notably lysine and methionine, cannot be synthesized by humans or animals, and must therefore be acquired *via* food sources. However, imbalances in plant nutrition are often caused by a lack of certain EAAs (Galili et al., 2016). Amino acids also act as intermediates of final metabolites in certain metabolic pathways, as well as participating in the regulation of multiple metabolic pathways, and other physiological and biochemical pathways, thereby affecting numerous physiological processes in plants (Amir et al., 2018; Yang et al., 2018).

EAAs in plants have been studied extensively (Galili et al., 2016). Genetic engineering provides an effective way of improving EAA levels in plants, with numerous successful results so far, albeit with additional unexpected effects (Galili et al., 2016). Of all living organisms, higher plants are thought to exhibit the highest complexity in terms of metabolic networks, making metabolic engineering of compositional traits particularly challenging (Yang et al., 2018). This review provides an overview of the connections between amino acid metabolisms in plants, with particular focus on new discoveries that have been made in recent years. In-depth analysis of the metabolic connections between amino acids will deepen our understanding of the genetic basis of plant genomes and metabolomes, providing new information for breeding of high-EAA crops.

## Metabolic Flux of Amino Acids in Plants

Studies have shown that regulating metabolism of a certain amino acid affects the level of other amino acids. This is mainly due to the biosynthesis and catabolism of amino acids derived from the same metabolic trunk and/or closely related to other metabolic pathways, acting as a synthetic substrate or intermediate (Long et al., 2013; Song et al., 2013).

**Figure 1:**
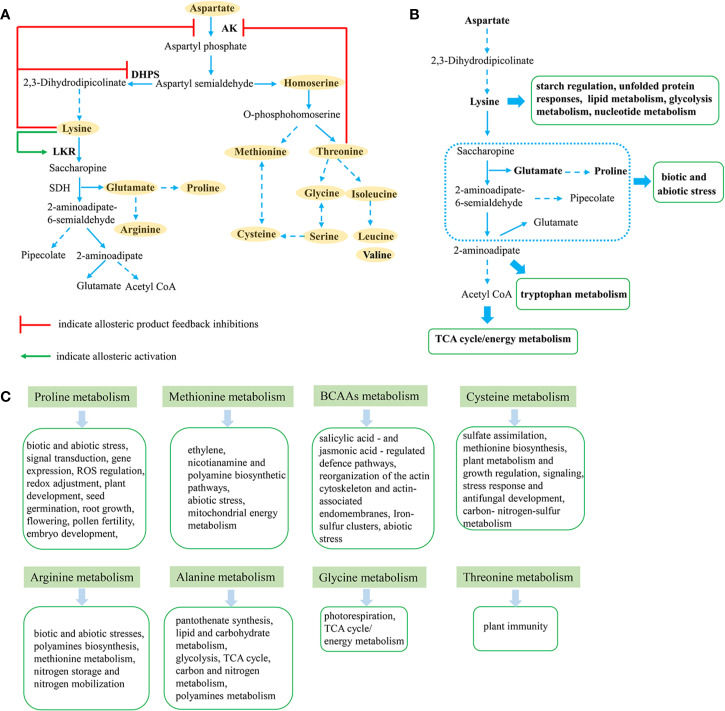
Metabolic connections between aspartate-derived amino acids in plants. **(A)** Aspartate-derived amino acids (highlighted in orange) metabolism pathway in plants. **(B)** Brief sketch of lysine metabolism and connected metabolic pathways (green box). **(C)** Aspartate-derived amino acids connected metabolic pathways (green box).

The aspartate family pathway leads to four key EAAs, lysine, methionine, threonine and isoleucine, but is also strongly associated with homoserine, glutamate, glycine, and proline (**Figure 1A**). Engineering of lysine metabolism tends to alter other amino acids in the aspartate family, even those from other pathways, suggesting a close regulatory network in the biosynthesis of free amino acids (Yang et al., 2018). Similar findings were found during the regulation of methionine metabolism (Song et al., 2013). Interestingly, high tryptophan levels were found to be associated with high-lysine plants (Yang et al., 2018; Wang et al., 2019), while Yang et al. (2018) revealed that the accumulation of lysine induced the tryptophan synthesis and metabolism in high free lysine transgenic rice (*Oryza sativa* L.). However, few reports have documented the relationship between metabolism of different amino acid families, highlighting the need for further research.

## Metabolic Connections between Lysine and Other Metabolites in Plants

The lysine metabolic pathways have been well elucidated, and enhanced accumulation of lysine has been achieved *via* metabolic engineering. Nevertheless, the potential connections with lysine fortification by metabolic engineering still need to be studied.

### Effects of Lysine Enrichment in Plants

Efforts have been made to increase the lysine content by altering lysine metabolism or expressing high-lysine protein in plants (Sun and Liu, 2004). However, seed germination in high-lysine Arabidopsis [*Arabidopsis thaliana* (L.) Heynh.] was found to be significantly retarded and reduced (Zhu and Galili, 2004). Moreover, in soybean [*Glycine max* (Linn.) Merr.], high seed-specific expression of feedback-insensitive genes encoding dihydrodipicolinate synthase (DHPS) and aspartate kinase (AK) was found to result in wrinkled seeds and low germination rates (Falco et al., 1995), while reduced grain yield was observed in high-lysine crops (Liu et al., 2016). Studies also suggest that lysine affects starch synthesis and/or endosperm development in maize (*Zea mays* Linn.) and rice *via* biofortification (Jia et al., 2013; Liu et al., 2016). The aggravated chalkiness phenotype was also found in the seed endosperm of transgenic rice and maize (Wong et al., 2015). We recently generated transgenic rice with elevated free lysine, revealing a slight decrease in isoleucine content and enhancement of other EAAs, with a significant increase in the tryptophan content (Yang et al., 2016). Yet, a dark-brown endosperm associated with the high free lysine phenotype was also revealed in engineered mature seeds (Yang et al., 2018). The above findings suggest that an excessive increase in lysine content also affects other metabolic pathways, thereby affecting growth and development of the target plant. However, these effects also differ between different species, suggesting that differences in the metabolic flux and connections with lysine metabolism also exist.

### Connections Between Lysine and Energy Metabolism

In addition to the classic role of aspartate acids in the synthesis of intracellular proteins, in the case of energy deficiency, lysine and isoleucine catabolism flux directly into the tricarboxylic acid (TCA) cycle, while threonine and methionine are converted into isoleucine (**Figure 1A**) (Wang et al., 2018). A negative effect on seed germination was also observed in high free lysine transgenic Arabidopsis expressing the *Escherichia coli dhps* gene in the *lkr* mutant (Angelovici et al., 2011). DHPS and lysine ketoglutaric acid reductase (LKR) represent two key enzymes involved in lysine biosynthesis and catabolism, respectively. Meanwhile, the results of metabolome and transcriptome analysis suggested significant increases in metabolites such as citrate, succinate, malate, and fumarate, which are involved in the TCA cycle, and the transcript of the gene encoding 2-oxoisovalerate dehydrogenase, which is associated with isoleucine catabolism in the TCA cycle, in germinated seeds with a high free lysine level, potentially affecting energy metabolism in the germinated seeds and subsequent seedling establishment (Angelovici et al., 2011). In addition, the glycine level also increased in high lysine plants, and was found to be the key intermediate metabolite during photorespiration (Schulze et al., 2016). Moreover, transcriptome analysis also revealed that the expression of photosynthesis- and photorespiration-associated genes was attenuated during seed germination in high lysine Arabidopsis (Angelovici et al., 2009; Angelovici et al., 2011).

### Connection Between Lysine Metabolism and Plant Stress Response

Lysine metabolism is involved in the plant stress response in various forms. It is mainly catabolized through the saccharopine pathway, which has been shown to play a role in abiotic and biotic stress responses (**Figures 1B, C**) (Kiyota et al., 2015; Bernsdorff et al., 2016; Arruda and Barreto, 2020). Moreover, under salt and osmotic stress, *LKR*/*SDH* expression was found to increase, while downstream metabolite pipecolate was enhanced (Kiyota et al., 2015). Furthermore, pipecolate was also found to increase significantly in Arabidopsis infected with pathogenic bacteria, playing a role in plant defense responses (Bernsdorff et al., 2016).

An increase in proline is also induced by glutamate from the saccharopine pathway and in response to osmotic and salt stress (Batista-Silva et al., 2019). When tissues or cells are under osmotic stress, α-aminoadipic semialdehyde dehydrogenase (AASADH), the third enzyme in the saccharopine pathway, is significantly up-regulated, suggesting that AASADH is also related to the osmotic stress response (Brocker et al., 2010). However, in maize, the saccharopine pathway is induced by exogenous lysine and repressed by salt stress, while proline and pipecolate synthesis are significantly repressed by lysine. While AASADH accumulates in tissues under salt, osmotic and oxidative stress, LKR/SDH enzyme is not produced (Kiyota et al., 2015). Meanwhile, in developing seeds, proline is thought to play a major role in abiotic stress responses in maize, similar to the role of pipecolate in Arabidopsis and canola (*Brassica napus* L.) (Kiyota et al., 2015). Beside this, proline plays multiple functions in other biotic and abiotic stresses, signal transduction, gene expression, ROS regulation, redox adjustment, and plant development (Trovato et al., 2019) (**Figure 1C**). We recently suggested that lysine metabolism induces the jasmonate signal pathway and tryptophan metabolism during stress responses, in contrast to the primary role of pipecolate, proline, and AASADH in other plant species (Yang et al., 2018). Briefly, while lysine catabolism is involved in the plant response to abiotic and biotic stress, there are obvious differences between plant species, in line with the differing stress responses of monocot and dicot plants.

Meanwhile, isoleucine, a branched chain amino acid (BCAA) that can also be induced by the aspartate pathway, plays a pivotal role in plant stress resistance as an osmo-regulation factor (Batista-Silva et al., 2019). Homoserine and threonine are also derived from the aspartate pathway, and the amino acid imbalances associated with homoserine and threonine accumulation were found to increase plant immunity to oomycete pathogens (Zeier, 2013).

### Metabolic Connections Between Lysine and Other Pathways

Starch, as an important source of carbohydrate, is also affected by lysine accumulation (Jia et al., 2013). In *opaque2* mutant maize, an increase in expression of several starch biosynthesis enzymes (GBSSI, Zpu1, SSIIa, BEI, BEIIb) was observed, leading to highly crystalline starch (Jia et al., 2013). Furthermore, Zhang et al. (2016) revealed that both *Opaque*-*2* and the prolamine-box binding factor network the regulation of protein and starch synthesis.

Unfolded protein responses (UPR) are characterized by the upregulation of chaperons and proteases, facilitating protein folding, and degrading unfolded protein (Ruberti and Brandizzi, 2018). In high-lysine rice with overexpression of exogenous proteins, BiP and PDI were induced in the endosperm, as well as being tightly linked to the floury/chalkiness grain phenotype, suggesting that lysine accumulation is also associated with UPR (Wong et al., 2015).

In canola and soybean, overexpression of the *Corynebacterium dapA* and *E. coli lysC* genes resulted in large increases in free lysine, but a decrease in oil production (Falco et al., 1995). Similar results have also been reported in maize (Medici et al., 2009). However, the high-lysine corn PQ15/CordapA, which was developed through reductions in lysine-poor seed storage proteins, zeins, and overexpressing *CordapA*, showed an increase in oil content in the grains (Huang et al., 2005). Furthermore, metabolome and transcriptome analysis showed a decrease in the lipid content in transgenic high lysine rice and up-regulation of mRNA levels of lipid metabolic genes (Angelovici et al., 2009; Angelovici et al., 2011; Yang et al., 2018). These findings suggest that the regulation of lysine metabolism affects lipid metabolism, but with differences between plant species.

During lysine production in microorganisms, cells produce a series of organic osmotic regulators, thereby maintaining normal osmotic pressure (Ying et al., 2014). Meanwhile, lysine production negatively interacts with acetate-associated metabolism during lysine fermentation (Anastassiadis, 2007). However, in rice and Arabidopsis, positive correlations between lysine accumulation and glycolysis metabolism were revealed (Angelovici et al., 2011; Yang et al., 2018).

Integrated metabolome and transcriptome analyses revealed that 12 genes encoding enzymes associated with nucleotide metabolism were stimulated, while levels of adenine, pseudouridine and uracil, which are related to nucleotide metabolism, were also improved in high-lysine plants (Angelovici et al., 2009; Yang et al., 2018). Lysine metabolism therefore affects the aspartate family pathway, which, in turn, may affect other metabolic pathways, confirming the complexity of the aspartate family pathway in plants.

## Connections Between Other Amino Acids and Their Related Metabolic Pathways

In general, amino acid metabolism is tightly linked to energy and carbohydrate metabolism, the carbon-nitrogen budget, and demands for protein synthesis and secondary metabolism (Pratelli and Pilot, 2014). Methionine is the main limiting sulfur EAA in plants, since it can be converted to cysteine in animals, thus meeting the requirements of both amino acids (Song et al., 2013). Both methionine and S-adenosylmethionine, participate in the ethylene, nicotianamine and polyamine biosynthetic pathways (Sauter et al., 2013). Moreover, the high methionine Arabidopsis was found to stimulate metabolic and transcriptomic responses associated with desiccation stress and mitochondrial energy metabolism (Cohen et al., 2014). A previous study also revealed that the higher the level of methionine in plants the higher their tolerance to abiotic stress (Ma et al., 2017). Cysteine is the first product of sulfate assimilation synthesized during the last stage of photosynthetic assimilation of sulfate (Gotor et al., 2015). It is not only a protein component, but also a source of methionine biosynthesis, and many other sulfur-containing metabolites are also involved in plant growth, signaling, stress responses, and antifungal development (Roblin et al., 2018; Kopriva et al., 2019). Furthermore, cysteine synthesis is the merging point of the three major pathways of carbon, nitrogen, and sulfur assimilation (Jobe et al., 2019).

The BCAAs isoleucine, valine and leucine are essential nutrients in humans and animals (Chen et al., 2010). BCAAs and their derivatives also contribute to plant growth, the stress response, and the production of food flavor components (Xing and Last, 2017). The BCAAs catabolism pathways have already been identified as essential during dehydration tolerance in Arabidopsis (Pires et al., 2016). They also appear to affect plant resistance to distinct pathogen classes by modulating crosstalk between salicylic acid- and jasmonic acid-regulated defense pathways (Zeier, 2013). Moreover, BCAA catabolism provides an alternative energy source under long-term dark treatment in plants (Peng et al., 2015). Recently, Cao et al. (2019) also revealed that BCAA over-accumulation leads to up-regulation of the target of rapamycin activity, which causes reorganization of the actin cytoskeleton and actin-associated endomembranes in Arabidopsis mutants. In addition, isoleucine was found to serve as a precursor for the synthesis of β-alanine in plants (Rouhier et al., 2019), while in Arabidopsis, a metabolic relationship was revealed between BCAA catabolism and iron-sulfur clusters *via* the mitochondrial homologue GRXS15 (Moseler et al., 2020).

Arginine and ornithine synthesize polyamines and are involved in plant responses to stresses. In addition, arginine constitutes a high percentage of the amino acid pool in storage proteins of conifers, while transcriptome and metabolome profiling also revealed significant genes and metabolites involved in arginine metabolism during late embryogenesis (Businge et al., 2012; Canales et al., 2014). Arginine metabolism is therefore thought to play a key role in nitrogen storage during embryogenesis and nitrogen mobilization during germination (Llebrés et al., 2018). Moreover, decarboxylated sadenosyl methionine from methionine metabolism serves as an aminopropyl donor during generation of polyamines (Gong et al., 2014). Evidence also suggests that plants synthesize β-alanine from spermine, uracil, and propionate (Parthasarathy et al., 2019). Similarly, metabolic tracing studies suggest that wheat synthesizes β-alanine from both isoleucine and propionate as in Arabidopsis (Reinhart and Rouhier, 2019). In plants, β-alanine is important for the synthesis of pantothenate, and subsequently coenzyme A, which is an essential coenzyme in lipid and carbohydrate metabolism (Parthasarathy et al., 2019). Glycolysis and the TCA cycle are linked by alanine aminotransferase during hypoxia induced by waterlogging (Rocha et al., 2010). Moreover, in rice, the alanine aminotransferase 1 encoded by the *Flo12* gene was found to simultaneously regulate carbon and nitrogen metabolism, while the *flo12* mutant presented a floury white-core endosperm (Zhong et al., 2019).

In plants, the aromatic amino acids phenylalanine, tyrosine, and tryptophan are not only essential components of protein synthesis, but are also located upstream of a number of growth hormones and secondary metabolites with multiple biological functions and health-promoting properties, such as protection against abiotic and biotic stress (Tzin and Galili, 2010). Phenylalanine is required for protein biosynthesis and cell survival (Tzin and Galili, 2010), and in plants, also acts as a precursor of a large number of multi-functional secondary metabolites. Among them, lignin is a principal structural component in the supporting tissues of vascular plants and some algae (Vanholme et al., 2019). Tyrosine is the central hub to a myriad of specialized metabolic pathways, while vitamin E and plastoquinone are essential metabolites of plant nutrition, photosynthesis, and antioxidant synthesis (Schenck and Maeda, 2018). Tyrosine is also a precursor of numerous specialized metabolites with diverse physiological roles such as non-protein amino acids, attractants, and defense compounds (Schenck and Maeda, 2018). Meanwhile, tryptophan is an essential EAA in the synthesis of a large number of bioactive molecules, such as auxin, tryptamine derivatives, phytoalexins, indole glucosinolates, and terpenoid indole alkaloids, as well as playing a pivotal role in the regulation of plant growth and development and stress responses. Recently, Accordingly, these findings have all been discussed extensively (Tzin and Galili, 2010; Hildebrandt et al., 2015; Datta et al., 2016).

Little is known about histidine metabolism and its connection with other amino acids in plants. A close correlation between nickel tolerance, root histidine concentration, and ATP-PRT transcript abundance was revealed in Hyperaccumulator plants, which show constitutively high expression of the histidine biosynthetic pathway (Ingle et al., 2005). Moreover, studies of *hisn1a* mutants revealed that histidine regulates seed oil deposition and protein accumulation *via* abscisic acid biosynthesis and β-oxidation in Arabidopsis (Ma and Wang, 2016). Histidine biosynthesis is tightly linked to nucleotide metabolism *via* 5′-phosphoribosyl 1-pyrophosphate, which is intermediate metabolite of anthranilate. These findings therefore suggest a metabolic link between histidine and tryptophan, nucleotide metabolism (Koslowsky et al., 2008).

## Future Prospectives

**Figure 2:**
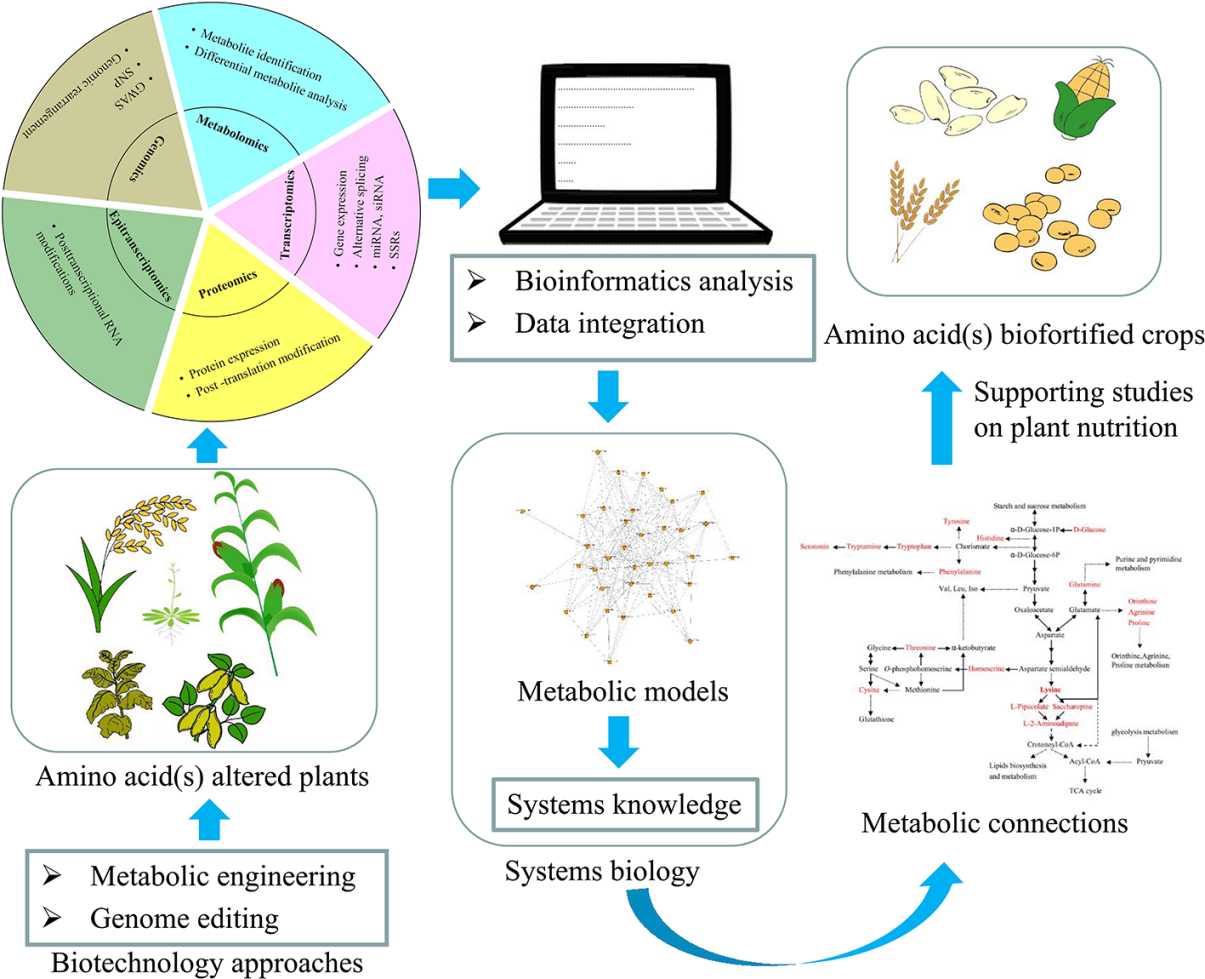
Schematic representation of the workflow depicting the application of different approaches to acquire a systems-level understanding of amino acid(s) metabolic connections in plants, and their application to obtain amino acid(s) biofortified crops.

Our knowledge of amino acid metabolism has increased exponentially in the past three decades. Amino acids and their derivatives have various prominent functions in plants, such as protein synthesis, growth and development, nutrition and stress responses (Hildebrandt et al., 2015). Meanwhile, metabolism is one of the most important and complex networks within biological systems, yet our understanding of metabolic regulation remains limited in terms of the modular operation of these networks. Precise and detailed information on biological and molecular mechanisms and metabolic connections is therefore essential. The recent development of omics approaches has been widely applied to studies of amino acid metabolisms and their connections (Gu et al., 2010; Angelovici et al., 2017; Xing and Last, 2017; Yang et al., 2018). Combinations of biochemistry, molecular genetics, genomics, and systems biology will continue to promote fundamental research, enabling us to develop ideas and strategies aimed at exploring new features of gene–protein–metabolite regulatory networks (**Figure 2**). Moreover, studies of epi-transcriptomics may provide a new strategy for analysis of metabolic connections in plants, giving insight into how different markers regulate a host of biological processes, from biosynthesis to catabolism and transport to function (Vandivier and Gregory, 2018). In addition, the continuously optimized gene editing technology of CRISPR-Cas has allowed the expression or activity of one or several key regulatory enzyme(s) to be altered, supporting studies aimed at improving the nutritional quality of plants (Chen et al., 2019).

In addition to technological issues, there are significant gaps in our knowledge of certain areas. Given the importance of the nutritional value of amino acids, the effects of amino acid (especially EAA) accumulation on other metabolic pathways during plant growth and development need further attention. To do so, analyses of the connections among amino acid metabolisms, transcriptional regulatory factors and post-translational modifications are essential. Thus, despite our growing knowledge of plant amino acid metabolisms and their metabolic connections, it is clear that many major discoveries have yet to be made.

## Author Contributions

QY and QL organized and wrote the manuscript. DZ provided critical evaluation and edited the text. All authors contributed to the article and approved the submitted version.

## Funding

This work was supported by the National Natural Science Foundation of China (31801322 and 31701393), the Ministry of Agriculture (2016ZX08001006-005), and the Government of Jiangsu Province (BE2018357 and PAPD), China.

## Conflict of Interest

The authors declare that this research was conducted in the absence of any commercial or financial relationships that could be construed as a potential conflict of interest.
